# Development of a standardised set of metrics for monitoring site performance in multicentre randomised trials: a Delphi study

**DOI:** 10.1186/s13063-018-2940-9

**Published:** 2018-10-16

**Authors:** Diane Whitham, Julie Turzanski, Lucy Bradshaw, Mike Clarke, Lucy Culliford, Lelia Duley, Lisa Shaw, Zoe Skea, Shaun P. Treweek, Kate Walker, Paula R. Williamson, Alan A. Montgomery, On behalf of the Site Performance Metrics for Multicentre Randomised Trials Collaboration, Simon Bevan, Lucy Bradshaw, Mike Clarke, Lucy Culliford, Adam Devall, Lelia Duley, Kathryn Fairbrother, Kirsteen Goodman, Catherine Hewitt, Rachel Hobson, Sarah Lawton, Stephen Lock, Alison McDonald, Alan Montgomery, John Norrie, Alastair O’Brien, Sarah Pearson, Shelley Rhodes, Lisa Shaw, Zoe Skea, Claire Snowdon, Kim Thomas, Shaun Treweek, Julie Turzanski, Kate Walker, Diane Whitham, Paula Williamson, Jill Wood

**Affiliations:** 1 Nottingham Clinical Trials Unit, Queens Medical Centre, C Floor, South Block, Nottingham, NG7 2UH UK; 2 School of Medicine, Dentistry and Biomedical Sciences, Institute of Health Sciences, Queen’s University, Belfast, UK; 3 CTEU Bristol, University of Bristol, Bristol Royal Infirmary, Bristol, BS2 8HW UK; 4 Institute of Neuroscience, Newcastle University, 3-4 Claremont Terrace, Newcastle upon Tyne, NE2 4AE UK; 5 Health Services Research Unit, University of Aberdeen, Health Sciences Building, Foresterhill, Aberdeen, AB25 2ZD UK; 6 MRC North West Hub for Trials Methodology Research, Department Biostatistics, University of Liverpool, Block F Waterhouse Building, 1-5 Brownlow Street, Liverpool, L69 3GL UK

**Keywords:** Multicentre randomised trials, Performance metrics, Delphi survey, Consensus meeting, Trial management

## Abstract

**Background:**

Site performance is key to the success of large multicentre randomised trials. A standardised set of clear and accessible summaries of site performance could facilitate the timely identification and resolution of potential problems, minimising their impact.

The aim of this study was to identify and agree a core set of key performance metrics for managing multicentre randomised trials.

**Methods:**

We used a mixed methods approach to identify potential metrics and to achieve consensus about the final set, adapting methods that are recommended by the COMET Initiative for developing core outcome sets in health care.

We used performance metrics identified from our systematic search and focus groups to create an online Delphi survey. We invited respondents to score each metric for inclusion in the final core set, over three survey rounds. Metrics scored as critical by ≥70% and unimportant by <15% of respondents were taken forward to a consensus meeting of representatives from key UK-based stakeholders. Participants in the consensus meeting discussed and voted on each metric, using anonymous electronic voting. Metrics with >50% of participants voting for inclusion were retained.

**Results:**

Round 1 of the Delphi survey presented 28 performance metrics, and a further six were added in round 2. Of 294 UK-based stakeholders who registered for the Delphi survey, 211 completed all three rounds.

At the consensus meeting, 17 metrics were discussed and voted on: 15 metrics were retained following survey round 3, plus two others that were preferred by consensus meeting participants. Consensus was reached on a final core set of eight performance metrics in three domains: (1) recruitment and retention, (2) data quality and (3) protocol compliance. A simple tool for visual reporting of the metrics is available from the Nottingham Clinical Trials Unit website.

**Conclusions:**

We have established a core set of metrics for measuring the performance of sites in multicentre randomised trials. These metrics could improve trial conduct by enabling researchers to identify and address problems before trials are adversely affected. Future work could evaluate the effectiveness of using the metrics and reporting tool.

**Electronic supplementary material:**

The online version of this article (10.1186/s13063-018-2940-9) contains supplementary material, which is available to authorized users.

## Background

Large multicentre randomised trials are complex projects. A key risk to their successful delivery is the performance of trial sites in recruiting and retaining participants, and in collecting complete high-quality data in a timely manner. Standardising the collection, reporting and monitoring of data relevant to site performance has the potential to improve the effective and efficient oversight of trial conduct [1–4].

Numerous variables or performance metrics can be measured to assess site performance. Measures of site performance should deliver meaningful, actionable information that can be compared within and between sites to initiate remedial action if necessary. A standardised set of clear and easily accessible summaries of site performance could facilitate the timely identification and resolution of problems, minimising their impact. Although researchers monitor data such as participant accrual, case report form returns, data quality, missing outcome data and serious protocol violations or breaches of good clinical practice, to our knowledge, no work has been conducted to establish a consensus on a core set of metrics for monitoring performance of sites in non-commercial clinical trials. Without a consensus, researchers may focus on too many or uninformative indicators. To be manageable and retain focus on items that really matter, a standardised set of site performance metrics would ideally number around eight to 12 items [1], and would be presented within a tool that could be easily monitored by a trial manager.

The aim of this study was to develop a standardised set of metrics for monitoring the performance of sites following their initiation and opening to patient recruitment in multicentre randomised trials. A further objective was to develop a visual display tool for reporting metric data.

## Methods

We used three focus groups of stakeholders (paper in preparation) and a systematic literature review to identify site performance metrics [5]. To achieve consensus on the final standardised set of metrics, we used a two-stage Delphi process comprising a survey followed by a consensus meeting of UK-based stakeholders.

### Delphi Survey

We identified 117 performance metrics from 21 eligible studies in the systematic literature review. Following initial analysis, we excluded 30 metrics judged as lacking clarity, unrelated to individual site performance, too specific to an individual trial methodology or pertaining to clinical outcomes rather than trial performance (Additional file 1). This left 87 for further consideration. The 32 participants in the three focus groups identified a further 19 metrics. Following deduplication and further removal of metrics considered unrelated to site performance, the remaining list of 28 metrics (Additional file 2) were organised into four thematic domains: (1) recruitment and retention, (2) data quality, (3) protocol compliance and (4) staff. These were used to create an online Delphi survey using the software COMET Delphi Manager [6].

#### Panel size and membership

As there is no standard method for calculating the sample size for Delphi processes, we used a pragmatic approach based on practicality and time available [2, 4]. The aim was to recruit the largest panel possible, encouraging individuals from each stakeholder group to participate via email invitations to the online survey. The stakeholder groups were:

- chief investigators
- members of the UK Clinical Research Network
- clinical trials unit (CTU) directors
- representatives of the main UK clinical trial funding bodies
- operations managers and directors
- clinical trial quality assurance managers
- research associates, fellows and academics
- research delivery managers
- trial managers and coordinators
- sponsors
- statisticians
- trial steering committee members

#### Recruitment of the panel

Clinical trials researchers were contacted through the UK Clinical Research Collaboration CTU Network and the UK Trial Managers’ Network. Representatives of the National Institute of Health Research (NIHR, a major funder of UK clinical trials), sponsors, chief investigators and UK Clinical Research Network representatives were identified through members of the project team, key contacts within the NIHR and the Trial Conduct Working Group of the Medical Research Council. The survey was also publicised on the Trial Forge website [7] and in a poster presentation at the 4th International Clinical Trials Methodology Conference [8]. Respondents were asked to complete the survey individually and to share the invitation with interested colleagues. Criteria for eligibility to complete the survey were being based in the UK and having at least three years’ experience of working in clinical trials.

#### Distributing the Delphi survey

An email invitation to the three-round Delphi survey contained a brief explanation of the study, emphasising the importance of completing all three rounds [3], an estimate of the time needed to complete each round (15 min) and a hyperlink to register with the survey. We aimed to complete each survey round within four weeks. Non-responders were sent automated reminders after one and two weeks, and a personalised email at the end of week 3. Rounds were extended by a few days if requested by participants to enable completion. Respondents were informed they would be entered into a prize draw if they completed all three rounds.

Upon registration, participants were asked to confirm that they were based in the UK and had at least three years’ experience working in clinical trials. They were asked to give their geographical region in the UK and their primary professional role. Participants’ names and contact details were recorded so that personalised reminders to complete the survey could be sent. However, the survey software prevented any individual survey responses being linked to individual names or contact details.

#### Conducting the Delphi survey

One thematic domain was presented per question page. Participants were asked to score each metric according to the importance of including it in a core set of essential metrics for monitoring the performance of sites during a trial. The Grading of Recommendations Assessment Development and Evaluation (GRADE) scale was used, which suggests a 9-point Likert scale (1 to 9) to rank importance [4]. Scores of 7 to 9 denote metrics of critical importance, scores of 4 to 6 are important but not critical, and scores of 1 to 3 are deemed not important. An option for unable to score (10) and a space to provide optional feedback on reasons for allocating particular scores were included. Participants could nominate additional metrics in round 1 to be included in round 2. New metrics were added to the list for round 2 if two or more participants suggested its inclusion, and it was not deemed to duplicate or overlap significantly with any other metric already in the survey [9].

Respondents were considered as a single panel. All round 1 metrics were carried forward to subsequent rounds. In rounds 2 and 3, each participant was presented with the distribution of scores from all participants in the previous round alongside their own score for each metric. Participants were asked to consider the responses from the other participants and review their score, either confirming or changing it. A space was provided for participants to explain their reasons for changing an individual score. Invitation to participate in rounds 2 and 3 was contingent upon completing the preceding round, as participants were always presented with their own scores from the previous round.

To investigate potential attrition bias [4, 10], we compared round 1 item mean scores and the percentage of respondents scoring each metric as critical for participants who completed only round 1 with those of participants who went on to complete round 2. We similarly compared round 2 data for participants who completed only rounds 1 and 2 with those participants who went on to complete round 3.

### Consensus criteria

We used the definitions of consensus described in Table 1 [4, 11]. Inclusion of an item in the subset to be discussed at the consensus meeting required agreement by the majority of survey participants regarding the critical importance of the metric, with only a minority considering it unimportant.

**Table 1:** Definition of consensus

Consensus classification	Description	Definition
Consensus in	Consensus that the metric should be included in the performance metric set	≥70% participants scoring 7 to 9 and < 15% participants scoring 1 to 3
Consensus out	Consensus that the metric should not be included in the performance metric set	≥70% participants scoring 1 to 3 and < 15% of participants scoring 7 to 9
No consensus	Uncertainty about the importance of the metric	Anything else

Source: [11]

#### Consensus meeting

Representatives of UK-based stakeholder groups and members of the study team were invited to attend a consensus meeting (September 2017). Prior to the meeting, we sent participants summary statistics for all 34 metrics from the Delphi survey. Ahead of the consensus meeting, participants were asked to review all the metrics that had reached the consensus in status following the survey, as only these metrics would be discussed and voted on during the meeting. Anyone wishing to make a case for discussion of any of the remaining metrics were given the opportunity to do so before the start of the meeting. At the consensus meeting, each metric was discussed in turn, and participants voted for its inclusion in the final core set using an anonymous electronic voting system. Metrics with >50% of participants voting for its inclusion were retained.

## Results

### Delphi Survey

**Fig. 1:**
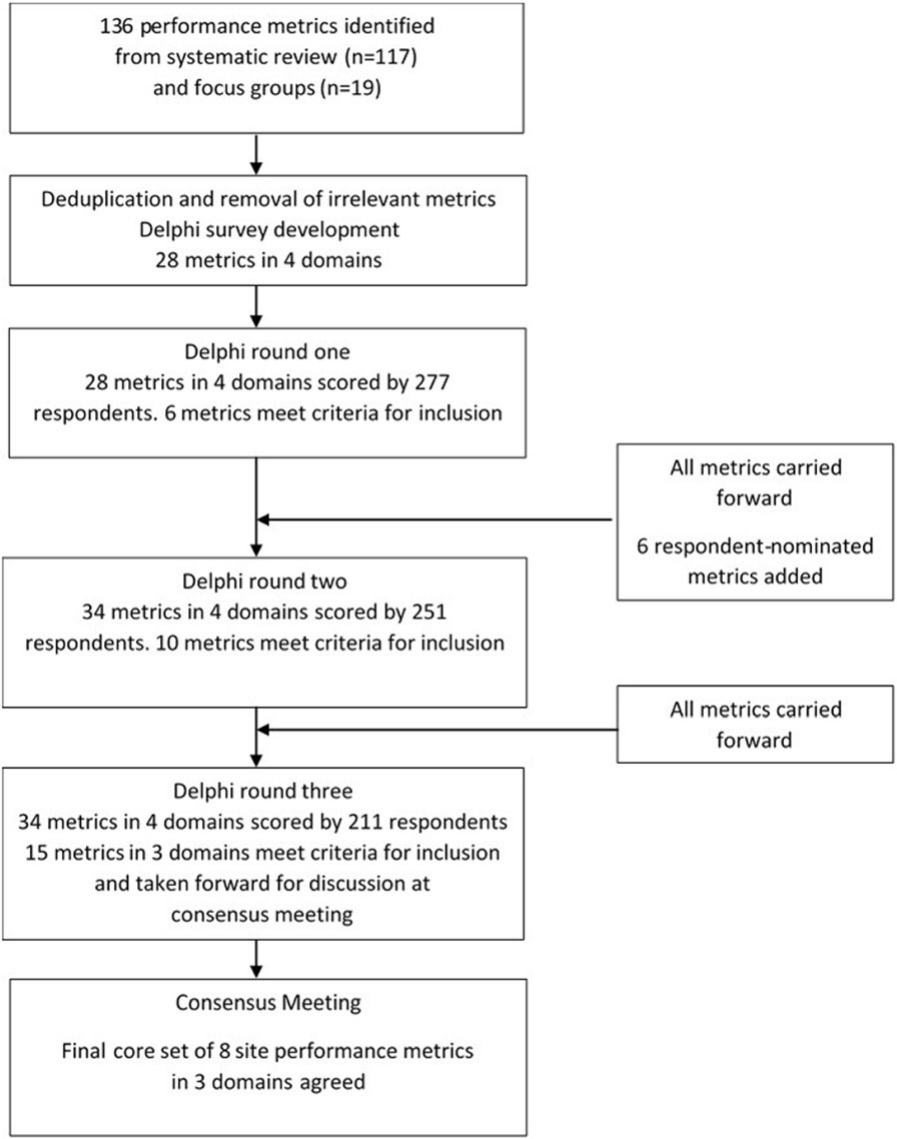
Summary results of Delphi survey and consensus meeting

Figure 1 summarises the Delphi study. Data were collected for the three rounds of the Delphi survey between June and September 2017. Of 294 people who registered for the survey, 277/294 (94%) completed round 1, 251/277 (91%) completed round 2 and 211/277 (76%) completed round 3. The within-round completion rate for round 3 was 211/251 (84%). Of the original 294, 280 (95%) had at least three years’ experience of working in clinical trials.

Table 2 shows the participation in each round of the Delphi survey by stakeholder group. Some participants represented more than one group, but are described here in their main role. Over half of all participants were involved in trial management (senior trial manager, project lead, manager, trial coordinator, or trial or research manager). The next largest group was chief investigators (13% in round 3). Although 66 participants who completed round 1 did not complete round 3, attrition appeared to be reasonably proportionate across all the stakeholder groups. Of 277 participants who completed round 1, 263 (95%) reported having at least three years’ experience working in clinical trials, compared with 200/211 (95%) who completed all three rounds. There was no evidence of attrition bias between rounds in terms of differences in metric scores between participants who did or did not complete subsequent survey rounds (Additional files 3 and 4).

**Table 2:** Delphi survey participation by stakeholder group

Role	All Registered		Round 1		Round 2		Round 3		
	*n*	% of total	*n*	% of total	*n*	% of total	*n*	% of total	% retained from round 1
Senior trial manager, project lead or manager	56	19.0	52	18.8	49	19.5	40	19.0	77
Trial or research manager	52	17.7	51	18.4	46	18.3	40	19.0	78
Trial coordinator	48	16.3	44	15.9	37	14.7	26	12.3	59
Chief investigator	34	11.6	32	11.6	29	11.6	27	12.8	84
Academic or research associate or fellow	18	6.1	18	6.5	17	6.8	16	7.6	89
Statistician	18	6.1	17	6.1	16	6.4	14	6.6	82
Operations manager or director	14	4.8	13	4.7	13	5.2	10	4.7	77
UK Clinical Research Network member	12	4.1	12	4.3	11	4.4	9	4.3	75
Clinical Trials Unit director	8	2.7	7	2.5	6	2.4	5	2.4	71
Quality assurance manager	8	2.7	8	2.9	8	3.2	8	3.8	100
Research delivery manager	4	1.4	3	1.1	2	0.8	2	0.9	67
Funder	2	0.7	2	0.7	2	0.8	2	0.9	100
Other	20	6.8	18	6.5	15	6.0	12	5.7	67
Total	294	100	277	100	251	100	211	100	76

The geographical region providing the largest group of participants who completed all three rounds was the East Midlands (22%), followed by London (15%) and the North-West England (12%). Other responses in round 3 were from: South-East England (10%), Scotland (8%), South-West England (8%), Yorkshire and Humber (6%), West Midlands (6%), North-East England (5%), Wales (4%) and Northern Ireland (1%).

Table 3 summarises the scores for each metric by Delphi survey round for the 211 participants who completed all three rounds and the outcome of the consensus meeting.

**Table 3:** Summary of item scores by survey round and outcome of consensus meeting

Metric and domain		Round 1 scores				Round 2 scores				Round 3 scores				% of consensus meeting participants voting to retain item
		1–3	4–6	7–9	10	1–3	4–6	7–9	10	1–3	4–6	7–9	10	
		*n* (%) respondents per rating category (1-3 ‘not important’, 4-6 ‘important but not critical’, 7-9 ‘critical’, 10 unable to score)												
Recruitment and retention	1. Total actual recruitment versus total target recruitment ^a,c^	1 (0.5)	36 (17.1)	172 (81.5)	2 (0.9)	0	16 (7.6)	194 (91.9)	1 (0.5)	0	14 (6.6)	197 (93.4)	0	100
	2. Time from the site opening to first participant randomised	8 (3.8)	108 (51.2)	94 (44.5)	1 (0.5)	3 (1.4)	118 (55.9)	90 (42.7)	0	5 (2.4)	126 (59.7)	80 (37.9)	0	
	3. Number of days/weeks since the most recent participant was randomised	21 (10.0)	112 (53.1)	77 (36.5)	1 (0.5)	12 (5.7)	142 (67.3)	57 (27.0)	0	11 (5.2)	155 (73.5)	45 (21.3)	0	
	4. Percentage of potential participants screened who have been randomised	4 (1.9)	88 (41.7)	117 (55.5)	2 (0.9)	2 (0.9)	83 (39.3)	125 (59.2)	1 (0.5)	0	76 (36)	134 (63.5)	1 (0.5)	
	5. Percentage of potential participants who could have been screened, who were screened	–	–	–	–	13 (6.2)	97 (46.0)	92 (43.6)	9 (4.3)	10 (4.7)	90 (42.7)	103 (48.8)	8 (3.8)	
	6. Percentage of potential participants screened who were eligible	–	–	–	–	9 (4.3)	106 (50.2)	93 (44.1)	3 (1.4)	6 (2.8)	110 (52.1)	92 (43.6)	3 (1.4)	
	7. Percentage of potential participants eligible who have consented ^b,c^	–	–	–	–	8 (3.8)	81 (38.4)	119 (56.4)	3 (1.4)	3 (1.4)	77 (36.5)	128 (60.7)	3 (1.4)	95
	8. Percentage of potential participants who have consented and have been randomised^a^	–	–	–	–	5 (2.4)	71 (33.6)	131 (62.1)	4 (1.9)	2 (0.9)	57 (27)	150 (71.1)	2 (0.9)	35
	9. Percentage of randomised participants who have withdrawn consent to continue in the study^a,c^	8 (3.8)	76 (36.0)	125 (59.2)	2 (0.9)	4 (1.9)	60 (28.4)	147 (69.7)	0	4 (1.9)	46 (21.8)	161 (76.3)	0	83
	10. Percentage of randomised participants lost to follow-up^a^	10 (4.7)	59 (28.0)	140 (66.4)	2 (0.9)	3 (1.4)	38 (18)	169 (80.1)	1 (0.5)	3 (1.4)	24 (11.4)	183 (86.7)	1 (0.5)	22
	11. Percentage of screening logs returned on time out of all those that should have been returned	40 (19.0)	135 (64.0)	33 (15.6)	3 (1.4)	29 (13.7)	159 (75.4)	22 (10.4)	1 (0.5)	23 (10.9)	167 (79.1)	20 (9.5)	1 (0.5)	
	12. Percentage of screening items completed of those required	32 (15.2)	105 (49.8)	67 (31.8)	7 (3.3)	20 (9.5)	114 (54)	72 (34.1)	5 (2.4)	18 (8.5)	117 (55.5)	72 (34.1)	4 (1.9)	
	13. Percentage of randomised participants with a consent form that is incomplete or inaccurate^a^	11 (5.2)	51 (24.2)	148 (70.1)	1 (0.5)	8 (3.8)	31 (14.7)	172 (81.5)	0	9 (4.3)	14 (6.6)	187 (88.6)	1 (0.5)	13
	14. Percentage of all expected forms that have been received^a^					8 (3.8)	69 (32.7)	128 (60.7)	6 (2.8)	4 (1.9)	50 (23.7)	154 (73)	3 (1.4)	39
	15. Percentage of randomised participants with any issues or problems with consent^a^	10 (4.7)	68 (32.2)	129 (61.1)	4 (1.9)	6 (2.8)	53 (25.1)	150 (71.1)	2 (0.9)	4 (1.9)	34 (16.1)	169 (80.1)	4 (1.9)	26
	16. Percentage of randomised participants for whom documentation of consent is missing from their medical records^a^	15 (7.1)	69 (32.7)	123 (58.3)	4 (1.9)	9 (4.3)	47 (22.3)	154 (73)	1 (0.5)	7 (3.3)	31 (14.7)	172 (81.5)	1 (0.5)	0
Data quality	17. Percentage of randomised participants with the time between data collection and either data entry (electronic case report form) or central receipt of paper case report form within the target timeframe	12 (5.7)	129 (61.1)	66 (31.3)	4 (1.9)	8 (3.8)	156 (73.9)	45 (21.3)	2 (0.9)	7 (3.3)	170 (80.6)	32 (15.2)	2 (0.9)	
	18. Percentage of randomised participants with a query/queries for primary outcome data ^a,c^	4 (1.9)	59 (28.0)	145 (68.7)	3 (1.4)	3 (1.4)	36 (17.1)	170 (80.6)	2 (0.9)	4 (1.9)	23 (10.9)	182 (86.3)	2 (0.9)	65
	19. Percentage of randomised participants with query/queries for secondary outcome data	16 (7.6)	128 (60.7)	65 (30.8)	2 (0.9)	8 (3.8)	156 (73.9)	46 (21.8)	1 (0.5)	8 (3.8)	162 (76.8)	40 (19)	1 (0.5)	
	20. Time taken between sending a data query and resolution of the query	17 (8.1)	140 (66.4)	52 (24.6)	2 (0.9)	10 (4.7)	164 (77.7)	36 (17.1)	1 (0.5)	9 (4.3)	167 (79.1)	34 (16.1)	1 (0.5)	
	21. Percentage of randomised participants with complete data for primary and important secondary outcomes^a,c^	2(0.9)	44 (20.9)	163 (77.3)	2 (0.9)	1 (0.5)	20 (9.5%)	189 (89.6)	1 (0.5)	1 (0.5)	11 (5.2)	198 (93.8)	1 (0.5)	96
	22. Percentage of randomised participants with complete data	3 (1.4)	85 (40.3)	120 (56.9)	3 (1.4)	0	88 (41.7)	122 (57.8)	1 (0.5)	0	91 (43.1)	119 (56.4)	1 (0.5)	
	23. Percentage of unresolved serious adverse event queries > 30 calendar days from the date the query was generated ^a^	3 (1.4)	44 (20.9)	163 (77.3)	1 (0.5)	1 (0.5)	24 (11.4)	186 (88.2)	0	1 (0.5)	12 (5.7)	198 (93.8)	0	9
	24. Total number of adverse events and serious adverse events reported per number of randomised participants	32 (15.2)	101 (47.9)	74 (35.1)	4 (1.9)	19 (9)	130 (61.6)	59 (28.0)	3 (1.4)	17 (8.1)	138 (65.4)	53 (25.1)	3 (1.4)	
	25. Number of serious adverse events reported per number of randomised participants	25 (11.8)	84 (39.8)	98 (46.4)	4 (1.9)	16 (7.6)	90 (42.7)	102 (48.3)	3 (1.4)	15 (7.1)	102 (48.3)	91 (43.1)	3 (1.4)	
	26. Number of adverse events reported per number of randomised participants ^b,c^	40 (19)	106 (50.2)	60 (28.4)	5 (2.4)	27 (12.8)	136 (64.5)	45 (21.3)	3 (1.4)	24 (11.4)	148 (70.1)	36 (17.1)	3 (1.4)	81
Protocol compliance	27. Percentage of randomised participants with at least one protocol violation ^a,c^	6 (2.8)	78 (37)	124 (58.8)	3 (1.4)	1 (0.5)	64 (30.3)	145 (68.7)	1 (0.5)	0	47 (22.3)	163 (77.3)	1 (0.5)	76
	28. Percentage of randomised participants receiving allocated intervention as intended per protocol ^a,c^	2 (0.9)	48 (22.7)	158 (74.9)	3 (1.4)	0	19 (9.0)	191 (90.5)	1 (0.5)	0	11 (5.2)	199 (94.3)	1 (0.5)	100
	29. Number of missed visits per number of randomised participants ^a^	7 (3.3)	93 (44.1)	107 (50.7)	4 (1.9)	5 (2.4)	75 (35.5)	128 (60.7)	3 (1.4)	4 (1.9)	52 (24.6)	152 (72)	3 (1.4)	10
	30. Number of late visits per number of randomised participants	18 (8.5)	128 (60.7)	61 (28.9)	4 (1.9)	10 (4.7)	157 (74.4)	41 (19.4)	3 (1.4)	9 (4.3)	162 (76.8)	37 (17.5)	3 (1.4)	
	31. Number of critical or major audit findings per number of randomised participants ^a^	6 (2.8)	43 (20.4)	152 (72)	10 (4.7)	4 (1.9)	23 (10.9)	179 (84.8)	5 (2.4)	3 (1.4)	14 (6.6)	190 (90)	4 (1.9)	0
Staff	32. Number of contacts from site staff to the central trial team within a given time period	79 (37.4)	112 (53.1)	14 (6.6)	6 (2.8)	76 (36)	124 (58.8)	6 (2.8)	5 (2.4%)	81 (38.4)	120 (56.9)	6 (2.8)	4 (1.9)	
	33. Time between protocol amendment being sent and principal investigator sign-off	22 (10.4)	110 (52.1)	73 (34.6)	6 (2.8)	16 (7.6)	127 (60.2)	65 (30.8)	3 (1.4)	15 (7.1)	140 (66.4)	53 (25.1)	3 (1.4)	
	34. Cumulative number of staff included on the delegation of duties log	–	–	–	–	105 (49.8)	87 (41.2)	12 (5.7)	7 (3.3)	116 (55.0)	83 (39.3)	7 (3.3)	5 (2.4)	

1–3 not important, 4–6 important but not critical, 7–9 critical and 10 unable to score

Scores shown for the 211 participants who completed all three rounds of the Delphi survey. Cells containing a dash indicate metrics that were added at round 2 and therefore not scored in round 1

^a^Metrics (*n* = 15) reaching consensus in status after survey round 3 and taken forward to the consensus meeting

^b^ Metrics 7 and 26 were also discussed and voted on at the meeting

^c^Metrics receiving > 50% of the vote at the meeting and retained in the final set

#### Round 1

Six metrics (numbered 1, 13, 21, 23, 28 and 31 in Table 3) reached the criterion for consensus in in round 1. No metrics were assigned a consensus out score. All 28 original metrics were carried forward to round 2 and six new metrics were added after round 1, following participants’ nominations. These were in the domains recruitment and retention (metrics 5–8 and 14) and staff (metric 34).

#### Round 2

Ten metrics (numbers 1, 10, 13, 15, 16, 18, 21, 23, 28 and 31, Table 3) reached the criterion for consensus in in round 2. All 34 metrics were carried forward to round 3.

#### Round 3

Altogether, 15 metrics (numbers 1, 8, 9, 10, 13, 14, 15, 16, 18, 21, 23, 27, 28, 29 and 31, Table 3) in three domains achieved the criterion for consensus in by the end of round 3 and were taken forward to the consensus meeting. No metrics from the staff domain were taken forward to the meeting. The reasons that participants reported for changing their scores between rounds related to further reflection and being influenced by the scores of others. None of the metrics reached the criterion for consensus out in any of the three survey rounds.

### Consensus meeting

In total, 35 UK-based stakeholders were invited to the consensus meeting, of whom 20 accepted and 16 attended. Participants represented trial managers, data managers, statisticians, quality assurance managers, CTU directors, chief investigators, research fellows, research networks and research funders. In addition, nine members of the study team attended, of whom seven voted, giving a total of 23 voting participants.

In addition to the 15 metrics reaching the criterion for inclusion after round 3 of the Delphi survey, a further two metrics (numbers 7 and 26) were discussed and voted on at the meeting. This was because several participants expressed a preference for these when metrics 8 and 23 were considered. There was a high level of agreement among participants. Of the 17 metrics that were discussed, 13 received over 75% of votes for either inclusion or exclusion from the final set (Table 3).

Eight metrics were included in the final core set: three each in the domains recruitment and retention and data quality, and two in protocol compliance (Table 4). The final wording for some of the metrics or the expanded definitions were altered to improve clarity following discussion at the consensus meeting. Table 4 shows the final versions and a comparison with the original versions.

**Table 4:** Recommended core set of site performance metrics (*n* = 8) retained following the consensus meeting

Domain	Metric: original wording	Definition: original wording	Metric: amended wording	Definition: amended wording
Recruitment and retention	1. Total actual recruitment versus total target recruitment (%)	The actual number of participants recruited into the trial by the site, versus the target number that was contractually agreed with the site prior to the trial commencing	Current actual recruitment versus target recruitment (%)	The actual number of participants recruited into the trial by the site, at the time of monitoring, versus the target number that was contractually agreed with the site prior to the trial commencing
	7. Percentage of potential participants eligible who have consented	The percentage of potential participants who were eligible to participate in the trial and who consented to participate	Percentage of eligible individuals who have consented	The percentage of individuals who were eligible to participate in the trial and who consented to participate
	9. Percentage of randomised participants who have withdrawn consent to continue	The percentage of randomised participants who have withdrawn their consent to any further participation in the trial at the site. Collection of any further follow up data is therefore not attempted		
Data quality	18. Percentage of randomised participants with a query for primary outcome data	The percentage of randomised participants at the site for whom the central trial team has sent one or more queries relating to the primary outcome data back to the site staff		
	21. Percentage of randomised participants with complete data for primary and important secondary outcomes	The percentage of randomised participants at the site with outcome data complete for both the primary outcome and all the agreed important secondary outcomes	Percentage of expected participants with complete data for primary and important secondary outcomes	
	26. Number of adverse events reported per number of randomised participants	Number of Adverse Events reported per number of randomised participants at the site	Percentage of randomised participants with at least one adverse event reported	The percentage of randomised participants at the site who have reported at least one adverse event
Protocol compliance	27. Percentage of randomised participants with at least one protocol violation	The percentage of randomised participants at the site with any protocol violation/s, as defined by the protocol		
	28. Percentage of randomised participants receiving allocated intervention as intended per protocol	The percentage of randomised participants at the site who started the allocated intervention, as specified in the protocol	Percentage of randomised participants who started allocated intervention	

Numbering of metrics refers to their position in the Delphi survey and Table 3

### Reporting tool

**Fig. 2:**
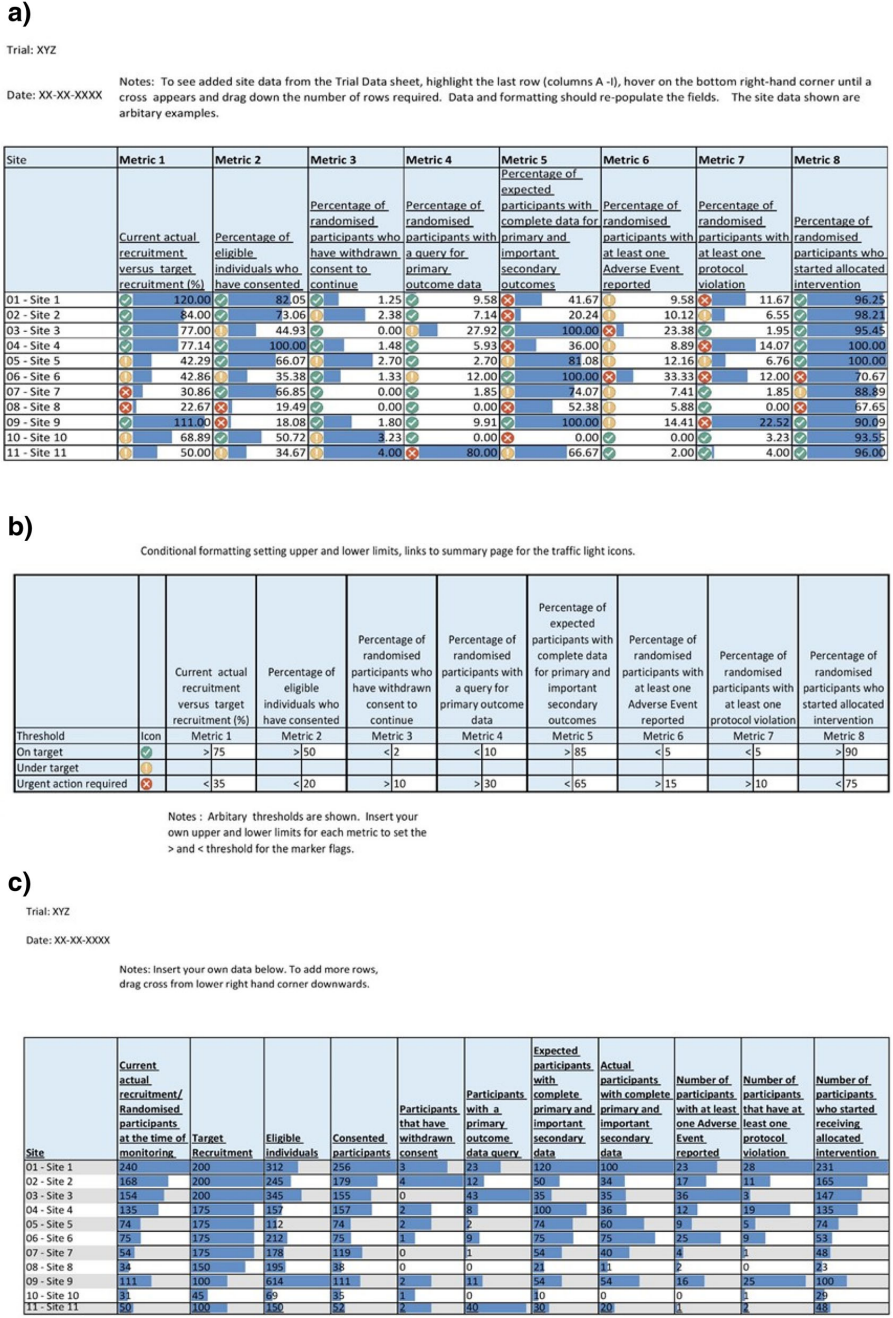
Worked example of site performance metrics reporting tool in Microsoft Excel. **a** Summary worksheet, **b** thresholds worksheet and **c** trial data worksheet

To support use of the core set of metrics, we have created a simple tool in Microsoft Excel, using a traffic light warning system to indicate potential problems (Fig. 2). The traffic light colours for each metric are linked to a set of thresholds. For example, when the percentage of participants with at least one protocol violation at a site is higher than 10%, this triggers a red traffic light. These thresholds are set by each trial team and may be quite different for different studies. The tool contains some default thresholds, but these are arbitrary and for illustration only. There are no accepted levels for any of them, although use of the tool may lead to some accepted values emerging. There may also be situations where a threshold changes during a trial. For example, an individual site’s current recruitment target could be reduced as the trial as a whole approaches its recruitment target and the certainty of meeting the overall sample size becomes clearer. The tool is freely available from the Nottingham Clinical Trials Unit website [12].

## Discussion

Using a mixed-methods approach, we achieved consensus on a core set of eight metrics for monitoring the performance of sites in multicentre randomised trials. The core set includes three metrics on recruitment and retention, three on data quality and two on protocol compliance. No metrics from the staff domain were included in the final set. To our knowledge, this is the first study that has attempted to identify a core set of key performance metrics for monitoring the conduct of clinical trials.

It is unsurprising that the number of participants recruited at sites was deemed critical for inclusion throughout the Delphi survey and supported unanimously at the consensus meeting. However, it is also notable that none of the 34 metrics achieved the criterion for consensus out in the survey, suggesting recognition by respondents that the ‘health’ of a multicentre randomised trial is multifaceted. Underlying problems with staff training, capacity, equipoise, integration of the trial into the clinical pathway or trial processes being inconvenient or time-consuming for participants could be reflected in several of the metrics included in the final set. If not addressed, these problems may affect patient safety, increase the risk of bias, or reduce the generalisability or statistical power.

Our study has several strengths. For the survey, we recruited a large sample of stakeholders with a wide range of roles in clinical trials from throughout the UK. This is important if the core set of metrics is to have credibility and relevance among potential users. Attrition in successive survey rounds diminishes group size. This can result in a false impression of how much consensus really exists [4], and may be due to participants losing interest, having insufficient time or holding minority views [13]. Over 75% of participants who completed round 1 went on to complete rounds 2 and 3, and there was no evidence of attrition bias, either in terms of different stakeholder groups or in mean scores of previous rounds. To facilitate use of the core set of performance metrics, we have developed a simple, user-friendly reporting tool in Microsoft Excel, which uses red, amber and green indicators based on thresholds for each metric, as determined by the trial team. This provides an at a glance performance check within and between trial sites, and could be used to complement existing trial management systems and data that are presented and discussed at regular trial management group meetings. Moreover, by using Excel, trials teams can modify the tool as they see fit to meet their own requirements.

### Limitations

There are also some limitations with our study. Although Delphi methods have been used successfully to develop core outcome sets and quality indicators in health-related research [2–4, 10, 14], there is no gold standard method for achieving consensus, and a different methodology may have produced a different final set of metrics [4, 15, 16].

Survey recruitment included a snowball sampling technique and participation was voluntary. Trial managers or those in similar roles made up the largest survey participant group, comprising half of the respondents who completed all three rounds. One could argue that this group have the greatest day-to-day role in monitoring site performance in multicentre randomised trials and therefore, should be strongly represented in the survey. However, even with half of survey participants in other roles, including senior positions, it is possible that the metrics selected for the consensus meeting reflect those considered most important by the dominant participant group.

Our focus was mainly on publicly funded trials led by academic researchers and our stakeholder representation reflects this focus. We believe that we obtained a broad and representative sample of UK-based stakeholders involved in these types of clinical trials. However, it is possible that another sample, for example with respondents from commercially led research, may have prioritised alternative metrics for inclusion.

Although we sought survey respondents who had been working in multicentre randomised trials for at least three years, a few participants who completed all three rounds indicated during survey registration that they did not have this level of experience. This was due to an error when we created the survey that allowed participants to proceed even if they reported not having at least three years’ experience in clinical trials. However, even if the length of experience is associated with which metrics are viewed as important, the small number of inexperienced participants is unlikely to have influenced the set taken forward to the consensus meeting.

As the Delphi survey is anonymous, there is no pressure for participants to conform. This may prevent those with strong views from dominating [3], but also means that conflicting views cannot be discussed or points explained [17, 18]. However, participants were able to provide feedback between rounds, and we made minor clarifications to the metric definitions in response. It is possible that participation in the consensus meeting by members of the research team may have been unintentionally influential in discussions, which may in turn have affected voting, although the meeting chairperson took care to invite and encourage wide discussion and did not permit individuals to dominate. Finally, we acknowledge the UK focus of this study and that other aspects of site performance may have greater importance in other settings.

## Conclusions

By using robust methods to achieve consensus, we have established a core set of eight metrics for measuring performance of sites in multicentre randomised trials. These metrics could improve trial conduct by helping researchers to identify and address problems at sites before trials are adversely affected. Future research should evaluate the effectiveness of using these core metrics in monitoring trial performance.

## Additional files

Additional file 1: Examples of site performance metrics excluded from the Delphi survey. (DOCX 21 kb)

Additional file 2: Site performance metrics (*n* = 34) and definitions included in the Delphi survey. (DOCX 19 kb)

Additional file 3: Examination of possible attrition bias in round 2 of the Delphi survey. (XLSX 18 kb)

Additional file 4: Examination of possible attrition bias in round 3 of the Delphi survey. (XLSX 18 kb)

## Abbreviations

CTU: Clinical trials unit
NIHR: National Institute for Health Research
SAE: Serious adverse event
