# Supplementary material for: Development of a standardised set of metrics for monitoring site performance in multicentre randomised trials: a Delphi study
Source: Trials. 2018 Oct 16;19:557. doi: 10.1186/s13063-018-2940-9 (PMC6192223; doi:10.1186/s13063-018-2940-9)
Supplement: Supplementary file 1 — Examples of site performance metrics excluded from the Delphi survey. (DOCX 21 kb) [file 13063_2018_2940_MOESM1_ESM.docx]

**Additional file 1: Examples of trial site performance metrics excluded from the Delphi survey**

| **Site performance metrics** | **Reason for exclusion** | **Study proposing metrics** |
| --- | --- | --- |
| Participant study progress | Lacking clarity | Kim J, Zhao W, Pauls K, Goddard T: **Integration of site performance monitoring module in web-based CTMS for a global trial**. *Clinical Trials* 2011, **8 (4)**:450 |
| Extent of staff  engagement/enthusiasm at a trial site | Lacking clarity/ difficult to express as a metric | Focus group suggestion |
| Number of missed visits per region | Unrelated to individual site performance | Berthon-Jones N, Courtney-Vega K, Donaldson A, Haskelberg H, Emery S, Puls R: **Assessing site performance in the Altair study, a multinational clinical trial**. *Trials* 2015, **16 (1) (no pagination)**(138) |
| Time from protocol release to ethics/regulatory submission | Trial set-up metric |  |
| Time from protocol release to ethics/regulatory approval | Trial set-up metric |  |
| Number of buffy coat samples collected versus protocol-mandated samples to be collected | Specific to an individual trial methodology |  |
| Site location potential index based on an assessment of the number of patients at an individual site with the disease of interest | Trial set-up metric | Bose A, Das S: **Trial analytics - A tool for clinical trial management**. *Acta Poloniae Pharmaceutica - Drug Research* 2012, **69**(3):523-533 |
| Timely database lock defined as time taken for database lock after the last visit of last participant per site | Not relevant for monitoring ongoing site conduct during a trial | Lee HJ, Lee S: **An Exploratory Evaluation Framework for e-Clinical Data Management Performance**. *Drug Information Journal* 2012, **46**(5):555-564 |
| Funding adjusted score = Intensity adjusted score (IAS) divided by the amount awarded for total direct costs during the given time period | Not relevant for monitoring ongoing site conduct during a trial | Rosendorf LL, Dafni U, Amato DA, Lunghofer B, Bartlett JG, Leedom JM, Wara DW, Armstrong JA, Godfrey E, Sukkestad E *et al*:  **Performance evaluation in multicenter clinical trials: Development of a model by the AIDS Clinical Trials Group**. *Controlled Clinical Trials* 1993, **14**(6):523-537 |
| Compliance with pain diaries | Refers to clinical outcome | Katz N: **Development and validation of a clinical trial data surveillance method to improve assay sensitivity in clinical trials**. *Journal of Pain* 2015, **1)**:S88 |
