# Supplementary material for: Development of a standardised set of metrics for monitoring site performance in multicentre randomised trials: a Delphi study
Source: Trials. 2018 Oct 16;19:557. doi: 10.1186/s13063-018-2940-9 (PMC6192223; doi:10.1186/s13063-018-2940-9)
Supplement: Supplementary file 2 — Site performance metrics (n = 34) and definitions included in the Delphi survey. (DOCX 19 kb) [file 13063_2018_2940_MOESM2_ESM.docx]

**Additional file 2:**

**Site performance metrics (n=34) and definitions included in the Delphi survey
n=28 metrics were taken from the systematic review and focus groups
n=6 (marked with *) metrics were added to the survey after Round 1**

| **Domain** | **Metric** | **Definition** |
| --- | --- | --- |
| Recruitment and retention | 1) Total actual recruitment versus total target recruitment | The actual number of participants recruited into the trial by the site, versus the target number that was contractually agreed with the site prior to the trial commencing |
| Recruitment and retention | 2) Time from the site opening to first participant randomised | The time between the date of greenlight approval for the site to start recruiting and the date the first participant was randomised |
| Recruitment and retention | 3) Number of days/weeks since the most recent participant was randomised | Number of days/weeks since the most recent participant at the site was randomised |
| Recruitment and retention | 4) Percentage of potential participants screened who have been randomised | The percentage of potential participants screened at the site who have been randomised |
| Recruitment and retention | *5) Percentage of potential participants who could have been screened, who were screened | The percentage of potential participants who could possibly have been screened, who were actually screened |
| Recruitment and retention | *6) Percentage of potential participants screened who were eligible | The percentage of potential participants who were screened and were eligible to participate in the trial |
| Recruitment and retention | *7) Percentage of potential participants eligible who have consented | The percentage of potential participants who were eligible to participate in the trial and who consented to participate |
| Recruitment and retention | *8) Percentage of potential participants who have consented and have been randomised | The percentage of potential participants who consented to take part in the trial and who have been randomised |
| Recruitment and retention | 9) Percentage of randomised participants who have withdrawn consent to continue in the study | The percentage of randomised participants who have withdrawn their consent to any further participation in the trial at the site. Collection of any further follow up data is therefore not attempted |
| Recruitment and retention | 10) Percentage of randomised participants lost to follow-up | The percentage of randomised participants at the site who are no longer responding to invitations to follow-up, and for whom no further attempt to follow-up is being made |
| Recruitment and retention | 11) Percentage of screening logs returned on time out of all those that should have been returned | Screening logs returned 'on time' means within the time period agreed with the site at the start of the trial, for example monthly screening data to be received no later than two weeks after the end of each month |
| Recruitment and retention | 12) Percentage of screening items completed of those required | The percentage of items on the site screening log that have been filled in out of all those required |
| Recruitment and retention | 13) Percentage of randomised participants with a consent form that is incomplete or inaccurate | The percentage of randomised participants at the site with a consent form that has either not been fully completed, or has been completed with inaccurate data |
| Recruitment and retention | *14) Percentage of all expected forms that have been received | The percentage of all expected documentation that has been received within a reasonable time frame |
| Recruitment and retention | 15) Percentage of randomised participants with any issues or problems with consent | The percentage of randomised participants at the site with any issues or problems with consent, including problems with the consent process (such as using the wrong version of the consent form or participant information sheet, or consent by someone not on the delegation log) as well as problems with completing the consent form |
| Recruitment and retention | 16) Percentage of randomised participants for whom documentation of consent is missing from their medical records | The percentage of randomised participants at the site for whom documentation of consent (such as a copy of the signed consent form) is missing from their medical records |
| Data quality | 17) Percentage of randomised participants with the time between data collection and either data entry (electronic case report form) or central receipt of paper case report form within the target timeframe | The percentage of randomised participants at the site for whom the time between data collection and either data entry (if an electronic case report form) or central receipt of the paper case report form is within the target timeframe |
| Data quality | 18) Percentage of randomised participants with a query/queries for primary outcome data | The percentage of randomised participants at the site for whom the central trial team has sent one or more queries relating to the primary outcome data back to the site staff |
| Data quality | 19) Percentage of randomised participants with query/queries for secondary outcome data | The percentage of randomised participants at the site for whom the central trial team has sent one or more queries relating to the secondary outcome data back to the site staff |
| Data quality | 20) Time taken between sending a data query and resolution of the query | The time from the central co-ordinating team sending a data query to the site (based on data they have received from the site) asking for further data or clarification, to a response that resolves that query |
| Data quality | 21) Percentage of randomised participants with complete data for primary and important secondary outcomes | The percentage of randomised participants at the site with outcome data complete for both the primary outcome and all the agreed important secondary outcomes |
| Data quality | 22) Percentage of randomised participants with complete data | The percentage of randomised participants at the site with complete data for the primary outcome and all the secondary outcomes |
| Data quality | 23) Percentage of unresolved Serious Adverse Event (SAE) queries > 30 calendar days from the date the query was generated | The percentage of queries about a Serious Adverse Event sent to the site from the central co-ordinating centre that remain unresolved more than 30 days after the query was sent |
| Data quality | 24) Total number of Adverse Events and Serious Adverse Events reported per number of randomised participants | The total number of Adverse Events and Serious Adverse Events reported per number of randomised participants at the site |
| Data quality | 25) Number of Serious Adverse Events reported per number of randomised participants | Number of Serious Adverse Events reported per number of randomised participants at the site |
| Data quality | 26) Number of Adverse Events reported per number of randomised participants | Number of Adverse Events reported per number of randomised participants at the site |
| Protocol/ compliance | 27) Percentage of randomised participants with at least one protocol violation | The percentage of randomised participants at the site with any protocol violation/s, as defined by the protocol |
| Protocol/ compliance | 28) Percentage of randomised participants receiving allocated intervention as intended per protocol | The percentage of randomised participants at the site who completed the allocated intervention, as specified in the protocol |
| Protocol/ compliance | 29) Number of missed visits per number of randomised participants | Number of missed visits per number of randomised participants at the site, where a missed visit is when a participant fails to complete a particular follow-up occasion |
| Protocol/ compliance | 30) Number of late visits per number of randomised participants | Number of late visits per number of randomised participants at the site, where a late visit is when a participant completes a particular follow-up occasion later than the agreed permissible time frame |
| Protocol/ compliance | 31) Number of critical or major audit findings per number of randomised participants | Number of critical or major audit findings, following a Good Clinical Practice (GCP) inspection, per number of randomised participants at the site |
| Staff | 32) Number of contacts from site staff to the central trial team within a given time period | Number of contacts from site staff (includes all communication from the site, for example by email or telephone) to the central trial team within a given time period |
| Staff | 33) Time between protocol amendment being sent and Principal Investigator sign-off | The time between a protocol amendment being sent by the central trial team and the signed agreement by the Principal Investigator to work to the amended protocol |
| Staff | *34) Cumulative number of staff included on the delegation of duties log | Number of staff included on the delegation of duties log since the study opened for recruitment at the site |
